# NLRP3 Is Involved in the Maintenance of Cerebral Pericytes

**DOI:** 10.3389/fncel.2020.00276

**Published:** 2020-08-21

**Authors:** Wenqiang Quan, Qinghua Luo, Qiqiang Tang, Tomomi Furihata, Dong Li, Klaus Fassbender, Yang Liu

**Affiliations:** ^1^Department of Clinical Laboratory, Tongji Hospital, Tongji University Medical School, Shanghai, China; ^2^Department of Neurology, Saarland University, Homburg, Germany; ^3^Department of Neurology, The First Affiliated Hospital of University of Science and Technology of China (Anhui Provincial Hospital), Hefei, China; ^4^Department of Clinical Pharmacy and Experimental Therapeutics, School of Pharmacy, Tokyo University of Pharmacy and Life Sciences, Tokyo, Japan

**Keywords:** Alzheimer’s disease, neuroinflammation, NLRP3, pericyte, cerebral perfusion

## Abstract

Pericytes play a central role in regulating the structure and function of capillaries in the brain. However, molecular mechanisms that drive pericyte proliferation and differentiation are unclear. In our study, we immunostained NACHT, LRR and PYD domains-containing protein 3 (NLRP3)-deficient and wild-type littermate mice and observed that NLRP3 deficiency reduced platelet-derived growth factor receptor β (PDGFRβ)-positive pericytes and collagen type IV immunoreactive vasculature in the brain. In Western blot analysis, PDGFRβ and CD13 proteins in isolated cerebral microvessels from the NLRP3-deficient mouse brain were decreased. We further treated cultured pericytes with NLRP3 inhibitor, MCC950, and demonstrated that NLRP3 inhibition attenuated cell proliferation but did not induce apoptosis. NLRP3 inhibition also decreased protein levels of PDGFRβ and CD13 in cultured pericytes. On the contrary, treatments with IL-1β, the major product of NLRP3-contained inflammasome, increased protein levels of PDGFRβ, and CD13 in cultured cells. The alteration of PDGFRβ and CD13 protein levels were correlated with the phosphorylation of AKT. Inhibition of AKT reduced both protein markers and abolished the effect of IL-1β activation in cultured pericytes. Thus, NLRP3 activation might be essential to maintain pericytes in the healthy brain through phosphorylating AKT. The potential adverse effects on the cerebral vascular pericytes should be considered in clinical therapies with NLRP3 inhibitors.

## Introduction

Brain pericytes wrapping around endothelial cells regulate various functions in the brain, which include blood-brain barrier (BBB) permeability, angiogenesis, and capillary hemodynamic responses (Sweeney et al., 2016). Pericytes express platelet-derived growth factor receptor β (PDGFRβ) and CD13. The binding of PDGFRβ with endothelial cells-released platelet-derived growth factor (PDGF)-B is essential for pericyte proliferation and integration into the blood vessel (Lindahl et al., 1997). CD13 promotes angiogenesis in hypoxic tissues, and response to stimulation of angiogenic growth factors, such as vascular endothelial growth factor, basic fibroblast growth factor, and transforming growth factor (Rangel et al., 2007). Deficiency of PDGFRβ decreases pericyte number, accumulates blood-derived fibrin/fibrinogen, reduces vasculature, and attenuates blood flow in the mouse brain, which finally leads to the white matter lesions characterized by loss of oligodendrocytes, demyelination, and axonal degeneration (Montagne et al., 2018). Growing evidence suggests that pericyte impairment mediates vascular dysfunction and contributes to the pathogenesis of Alzheimer’s disease (AD; Love and Miners, 2016). In the AD human brain, pericytes are lost in association with increased BBB permeability at a very early disease stage (Sengillo et al., 2013; Nation et al., 2019). In AD mouse models that overexpress Alzheimer’s precursor protein (APP) in neurons, the deletion of pericytes increases deposition of amyloid β peptide (Aβ) in both brain parenchyma and blood vessels, which potentially exaggerates cognitive deficits (Sagare et al., 2013). However, molecular mechanisms that regulate pericyte survival and activation in the brain are largely unknown.

Pericytes express pattern recognition receptors, such as Toll-like receptor 2 and 4 (TLR-2 and -4) and NACHT, LRR and PYD domains-containing protein 1 and 3 (NLRP-1 and -3; Guijarro-Muñoz et al., 2014; Leaf et al., 2017; Nyúl-Tóth et al., 2017). Cultured brain pericytes release cytokines and chemokines after being challenged with lipopolysaccharide (LPS), tumor necrosis factor (TNF)-α or *E. coli* infection (Kovac et al., 2011; Guijarro-Muñoz et al., 2014; Nyúl-Tóth et al., 2017). Cultured pericytes secret active interleukin (IL)-1β when they are intracellularly stimulated with LPS, although how NLRP3-contained inflammasome is activated remains unclear (Nyúl-Tóth et al., 2017). It is interesting to ask whether innate immune signaling regulates cell fate and functions of pericytes in the brain.

In AD research, NLRP3-contained inflammasome attracted great attention, as it is activated in AD brain and potentially mediates microglial inflammatory responses, exaggerates Aβ and Tau protein aggregation in APP or Tau-transgenic mice (Heneka et al., 2013; Venegas et al., 2017; Ising et al., 2019; Stancu et al., 2019). NLRP3 is considered as a promising therapeutic target for AD patients (Dempsey et al., 2017). However, the effects of NLRP3 activation on pericytes and vascular dysfunction were not addressed. The animal models used in published studies have also limited AD-associated vascular pathology. Thus, whether NLRP3 inhibition protects or damages microvascular circulation in the AD brain remains unclear. Moreover, AD pathology is mainly localized in temporal and parietal lobes, instead of covering the whole brain. Between AD lesion sites as shown with Aβ deposits, neurofibrillary tangles, and gliosis, the brain tissues are relatively or absolutely healthy (Deture and Dickson, 2019). It is, therefore, necessary to understand the physiological function of NLRP3 in brain pericytes, which is helpful to predict potential off-target effects of NLRP3 inhibitors in the future anti-AD therapies.

In this study, we used NLRP3-knockout mice and treated cultured pericytes with NLRP3 inhibitor, MCC950, or IL-1β, a major product of NLRP3-contained inflammasome. We observed that NLRP3 might be essential for the maintenance of healthy pericytes in the brain. We further observed that AKT (also known as protein kinase B) might mediate the physiological function of NLRP3 in pericytes.

## Materials and Methods

### Mice

NLRP3-encoding gene knockout (NLRP3^−/−^) mice were kindly provided by N. Fasel (University of Lausanne, Lausanne, Switzerland; Martinon et al., 2006). Mice with the knockout of gene encoding myeloid differentiation primary response 88 (MyD88^−/−^) were originally provided by S. Akira and K. Takeda (Osaka University, Osaka, Japan; Adachi et al., 1998). Breeding between heterozygous mutants (+/−) on a C57BL/6 background was used to maintain mouse colonies. Mice were compared only between littermates. Animal experiments were performed following all relevant national rules and were authorized by the local research ethics committee.

### Tissue Collection and Isolation of Blood Vessels

Animals were euthanized by inhalation of isoflurane and perfused with ice-cold phosphate-buffered saline. The brain was removed and divided. The left hemisphere was immediately fixed in 4% paraformaldehyde (Sigma-Aldrich Chemie GmbH, Taufkirchen, Germany) for immunohistochemistry. The cortex and hippocampus from the right hemisphere were carefully dissected and brain vessel fragments were isolated according to the published protocol (Boulay et al., 2015). Briefly, brain tissues were homogenized in HEPES-contained Hanks’ balanced salt solution (HBSS) and centrifuged at 4,400 *g* in HEPES-HBSS buffer supplemented with dextran from Leuconostoc spp. (molecular weight ~70,000; Sigma-Aldrich) to delete myelin. The vessel pellet was re-suspended in HEPES-HBSS buffer supplemented with 1% bovine serum albumin (Sigma-Aldrich) and filtered with 20 μm-mesh. The blood vessel fragments were collected on the top of the filter for biochemical analysis and stored at −80°C for biochemical analysis.

### Histological Analysis

To analyze pericytes in capillaries, serial 30-μm-thick sagittal sections were cut from the dehydrated and cryoembedded left brain hemisphere with a Leica cryostat (Leica Mikrosysteme Vertrieb, Wetzlar, Germany). Three sections per mouse with 300 μm of an interval between neighboring layers were used. Antigen retrieval was performed by heating sections in 10 μM citrate buffer (pH = 6). After blocking with 5% goat serum in PBS/0.3% Triton X-100, brain sections were incubated with rabbit anti-PDGFRβ monoclonal antibody (clone: 28E1; Cell Signaling Technology Europe, Frankfurt am Main, Germany) at 4°C overnight and then Alexa488-conjugated goat anti-rabbit IgG (Thermo Fisher Scientific, Darmstadt, Germany) at room temperature for 1 h. Thereafter, brain sections were further stained with biotin-labeled *Griffonia simplicifolia* Lectin I isolectin B4 (Catalog: B-1205; Vector Laboratories, Burlingame, CA, USA) and Cy3-conjugated streptavidin (Roche Applied Science, Mannheim, Germany). The whole cortex was imaged under a Zeiss AxioImager.Z2 microscope (Carl Zeiss Microscopy GmbH, Göttingen, Germany) equipped with a Stereo Investigator system (MBF Bioscience, Williston, VT, USA). Ten regions per section were randomly chosen under a 40× objective. Blood vessels with <6 μm of diameter were examined. Fluorescence-labeled areas were measured with ImageJ software^1^. The coverage of pericytes was calculated as a ratio of PDGFRβ/isolectin B4-positive staining area.

To detect the expression of NLRP3 in pericytes, brain sections were incubated at 4°C overnight with mouse anti-NLRP3 monoclonal antibody (clone: Cryo-2; AdipoGen Life Sciences, San Diego, CA, USA), which was followed by incubation with Cy3-conjugated goat anti-mouse IgG (Jackson ImmunoResearch Europe Ltd., Cambridgeshire, UK) at room temperature for 1 h. Thereafter, brain sections were further stained with rabbit anti-PDGFRβ monoclonal antibody and Alexa488-conjugated goat anti-rabbit IgG as described above. Stack images were acquired with a Zeiss AxioImager.Z2 microscope under a 63× oil objective with an interval of 0.2 μm between neighboring layers, processed with deconvolution and finally Z-projected with maximum intensity.

For analysis of the impairment of BBB in NLRP3-deficient mice, brain sections from NLRP3 - knockout and wild-type mice were stained with Alexa488-conjugated goat anti-mouse IgG (Thermo Fisher Scientific) after blocking with goat serum as we did in a previous study (Hao et al., 2011), and co-stained with biotinylated isolectin B4 and Cy3-conjugated streptavidin.

To quantify vasculature in the brain, our established protocol was used (Decker et al., 2018). The left hemisphere was embedded in paraffin and 40-μm-thick sagittal sections were serially cut. Four serial sections per mouse with 400 μm of distance in between were deparaffinized, heated at 80°C in citrate buffer (10 mM, pH = 6) for 1 h, and digested with Digest-All 3 (Pepsin; Thermo Fisher Scientific) for 20 min. Thereafter, brain sections were stained with rabbit anti-collagen IV polyclonal antibody (Catalog: #ab6586; Abcam, Cambridge, UK) and Alexa488-conjugated goat anti-rabbit IgG (Thermo Fisher Scientific). After being mounted, the whole brain including the hippocampus and cortex was imaged with Microlucida (MBF Bioscience). The length and branching points of collagen type IV staining-positive blood vessels were analyzed with free software, AngioTool^2^ (Zudaire et al., 2011). The parameters of analysis for all compared samples were kept constant. The length and branching points were adjusted with an area of interest.

### Western Blot Analysis

Isolated blood vessels were lysed in RIPA buffer [50 mM Tris (pH 8.0), 150 mM NaCl, 0.1% SDS, 0.5% sodium deoxycholate, 1% NP-40, and 5 mM EDTA] supplemented with protease inhibitor cocktail (Roche Applied Science) on ice. The tissue lysate was sonicated before being loaded onto 10% SDS-PAGE. For Western blot detection, rabbit monoclonal antibodies against PDGFRβ and CD13/APN (clone: 28E1 and D6V1W, respectively; Cell Signaling Technology Europe) were used. In the same sample, β-actin was detected as a loading control using a rabbit monoclonal antibody (clone: 13E5; Cell Signaling Technology Europe). Western blots were visualized *via* the ECL method (PerkinElmer LAS GmbH, Rodgau, Germany). Densitometric analysis of band densities was performed with ImageJ software. For each sample, the protein level was calculated as a ratio of target protein/β-actin.

### Culture of Pericytes

Human primary brain vascular pericytes (HBPC) were immortalized by infecting cells with tsSV40T lentiviral particles (Umehara et al., 2018). The selected immortalized HBPC clone 37 (hereafter referred to as HBPC/ci37) was used for our study. HBPC/ci37 cells were cultured at 33°C with 5% CO_2_/95% air in pericyte medium (Catalog: #1201; Sciencell Research Laboratories, Carlsbad, CA, USA) containing 2% (v/v) fetal bovine serum, 1% (w/v) pericyte growth factors, and penicillin-streptomycin. Culture flasks and plates were treated with Collagen Coating Solution (Catalog: #125-50; Sigma-Aldrich). HBPC/ci37 cells were used at 40–60 passages in this study.

### Analysis of Pericyte Proliferation and Apoptosis

Pericytes were seeded at 1.0 × 10^4^ cells on 96-well plate/100 μl (day 0), and cultured in pericyte medium containing NLRP3 inhibitor, MCC950 (Catalog: #PZ0280; Sigma-Aldrich), at 0, 25, 50 and 100 nM, or containing recombinant human IL-1β (Catalog: #201-LB; R&D Systems, Wiesbaden, Germany) at 0, 5, 10 and 50 ng/ml. The cell survival was detected with MTT-based Cell Proliferation Kit I (Catalog: #11465007001; Sigma-Aldrich) on days 1, 2, 3, 4, 5, 6 and 7. In MTT assay, yellow and water-soluble 3-(4,5-dimethylthiazol-2-yl)-2,5-diphenyl tetrazolium bromide (MTT) enters viable cells and passes into the mitochondria where it is reduced by succinate dehydrogenase to an insoluble, dark purple formazan product. The measured absorbance as shown with optical density (OD) at 590 nm is proportional to the number of viable cells.

To further detect cell death and proliferation of pericytes, cells were cultured in 12-well plate at 5.0 × 10^5^ cells/well, and treated with MCC950 as described in MTT assay. After 24 h, pericytes were collected and lysed in RIPA buffer. Quantitative Western blot was used with rabbit monoclonal antibody against cleaved caspase-3 (clone: 5A1E; Cell Signaling Technology Europe), mouse monoclonal antibody against proliferating cell nuclear antigen (PCNA; clone: PC10; Cell Signaling Technology Europe) and rabbit monoclonal antibody against Ki-67 (clone: SP6; Abcam). α-tubulin and β-actin were detected as internal control with mouse monoclonal antibody (clone: DM1A; Abcam) and rabbit monoclonal antibody (clone: 13E5; Cell Signaling Technology Europe), respectively.

In this experiment, we also detected NLRP3 and cleaved caspase-1 in the cell lysate from pericytes with and without treatment of MCC950, with rabbit monoclonal antibodies against NLRP3 and cleaved caspase-1 (Asp297; clone D4D8T and D57A2; Cell Signaling Technology Europe), respectively.

### Treatments of Pericytes for Detection of PDGFRβ and CD13 and Phosphorylated Kinases

Pericytes were cultured in a 12-well plate at 5.0 × 10^5^ cells/well. Before experiments, we replaced the culture medium with serum-free pericyte medium and cultured cells at 37°C for 3 days to facilitate cell differentiation (Umehara et al., 2018). Thereafter, pericytes were treated for 24 h with MCC950, at 0, 25, 50 and 100 nM, recombinant human IL-1β (Catalog: #201-LB; R&D Systems) at 0, 5, 10 and 50 ng/ml, or AKT Inhibitor VIII (Catalog: #124018; Sigma-Aldrich) at 0, 0.5, 1 and 5 μM. To investigate whether AKT mediates the effect of IL-1β, pericytes were pre-treated with 1 μM AKT inhibitor VIII for 1 h and then incubated with IL-1β at various concentrations in the presence of AKT inhibitor for 24 h. The cell lysate was prepared in RIPA buffer supplemented with protease inhibitor cocktail (Roche Applied Science) and phosphatase inhibitors (50 nM okadaic acid, 5 mM sodium pyrophosphate, and 50 mM NaF; Sigma-Aldrich). For quantitative Western blot, the following antibodies were used: rabbit monoclonal antibodies against PDGFRβ, CD13/APN, phosphorylated AKT (Ser473), phosphorylated ERK1/2 (Thr202/Tyr204), phosphorylated NFκB p65 (S536), NFκB p65, β-actin, GAPDH (clone: 28E1, D6V1W, D9E, D13.14.4E, 93H1, D14E12, 13E5, and 14C10, respectively; Cell Signaling Technology Europe), rabbit polyclonal antibodies against AKT and phosphorylated GSK-3β (Ser9; Catalog: #9272 and Catalog: #9336, respectively; Cell Signaling Technology Europe) and mouse monoclonal antibodies against ERK1/2 and GSK-3β (clone: L34F12 and 3D10, respectively; Cell Signaling Technology Europe) and α-tubulin (clone: DM1A; Abcam).

### Statistics

Data were presented as mean ± SEM for mice and mean ± SD for cells. For multiple comparisons, one-way or two-way ANOVA followed by Bonferroni or Tukey *post hoc* test. Two independent-samples Students *t*-test was used to compare means for two groups of cases. All statistical analyses were performed with GraphPad Prism 5 version 5.01 for Windows (GraphPad Software, San Diego, CA, USA). Statistical significance was set at *p* < 0.05.

## Results

### NLRP3 Deficiency Reduces Pericyte Cell Coverage and Decreases Protein Levels of PDGFRβ and CD13 in the Brain

**Figure 1:**
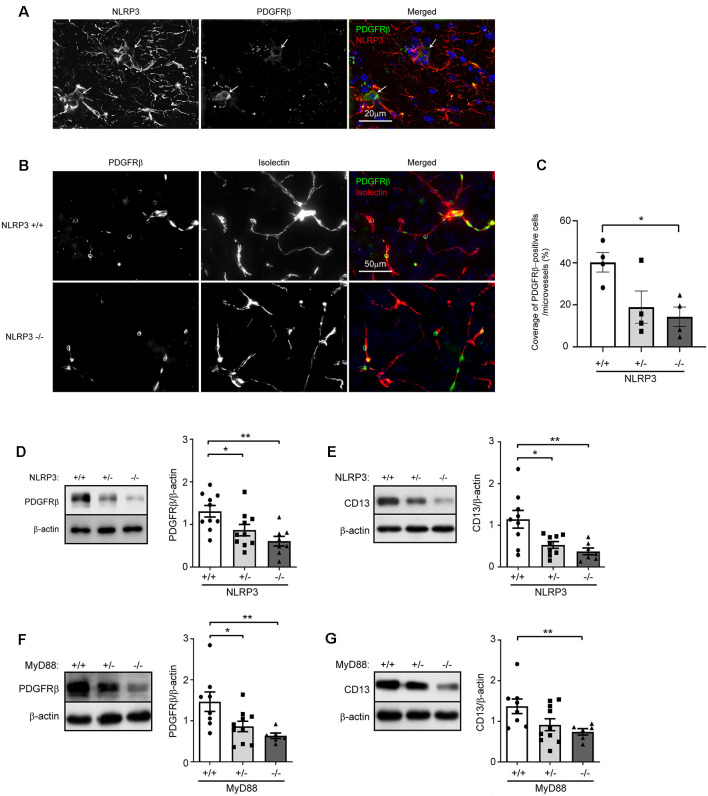
NLRP3 deficiency reduces pericyte cell coverage and decreases protein levels of PDGFRβ and CD13 in the brain. **(A)** Nine-month-old mouse brains were co-stained for NLRP3 and PDGFRβ. PDGFRβ-immune reactive cell bodies (in green; marked with arrows) were stained by NLRP3-specific antibodies (in red). **(B)** Brain tissues from 9-month-old NLRP3-knockout (−/− and +/−) and wild-type (+/+) littermate mice were then co-stained for PDGFRβ (with anti-PDGFRβ antibodies, in green) and endothelial cells (with isolectin B4, in red). **(C)** The coverage of PDGFRβ-positive pericytes was calculated as a ratio of PDGFRβ/isolectin B4-positive area. One-way ANOVA followed by Tukey *post hoc* test, *n* = 4 per group. **(D–G)** Nine-month-old NLRP3, and 6-month-old MyD88 littermate mice with homozygous (−/−) and heterozygous (+/−) knockout, and wild-type (+/+) of *nlrp3* and *myd88* genes, respectively, were analyzed for protein levels of PDGFRβ and CD13 in isolated cerebral blood vessels. One-way ANOVA followed by Bonferroni *post hoc* test, *n* = 10, 10 and 8 for NLRP3 (+/+, +/− and −/−) mice in PDGFRβ detection and *n* = 9, 9 and 7 for NLRP3 (+/+, +/− and −/−) mice in CD13 detection; *n* = 8, 10 and 6 for MyD88 (+/+, +/− and −/−) mice in the detection of both PDGFRβ and CD13. *p < 0.05 and **p < 0.01.

To explore the effects of NLRP3 on the maintenance of pericytes in the brain, we first detected the expression of NLRP3 in pericytes. In brain sections, we observed widely distributed NLRP3-immune reactive cell bodies and processes, part of which were co-stained by PDGFRβ-specific antibodies (Figure 1A), which suggests that pericytes express NLRP3. Then, we estimated the coverage of PDGFRβ-positive cells in brains from 9-month-old NLRP3-knockout (NLRP3^−/−^ and NLRP3^+/−^) and wild-type (NLRP3^+/+^) littermate mice. As shown in Figures 1B,C, deficiency of NLRP3 significantly decreased the coverage of PDGFRβ-immune reactive cells in microvessels with < 6 μm of diameter in a gene dose-dependent manner as compared with that in NLRP3-wildtype mice (one-way ANOVA, *p* < 0.05; *n* = 4 per group). We continued to isolate blood vessels from brains of 9-month-old NLRP3^−/−^, NLRP3^+/−^ and NLRP3^+/+^ littermate mice for the detection of pericyte protein markers, PDGFRβ, and CD13. We observed that the deletion of NLRP3 significantly reduced PDGFRβ and CD13 proteins in the cerebral blood vessels also in a gene dose-dependent manner (Figures 1D,E; one-way ANOVA, *p* < 0.05; *n* ≥ 7 per group). Unfortunately, we failed to detect cleaved caspase-3 and PCNA in the tissue lysate of blood vessels (data not shown), which are markers for cell apoptosis and cell proliferation, respectively.

In further experiments, we asked whether innate immune signaling serves a common effect on pericyte survival in the brain. We detected PDGFRβ and CD13 proteins in cerebral blood vessels isolated from 6-month-old MyD88^−/−^, MyD88^+/−^ and MyD88^+/+^ littermate mice. MyD88 is a common adaptor down-stream to most TLRs and mediates the inflammatory activation of IL-1β (O’Neill and Bowie, 2007). We observed that protein levels of CD13 and PDGFRβ were significantly lower in MyD88-deficient mice than in MyD88-wildtype controls (Figures 1F,G; one-way ANOVA, *p* < 0.05; *n* ≥ 6 per group).

### NLRP3 Deficiency Reduces Vasculature in the Brain

**Figure 2:**
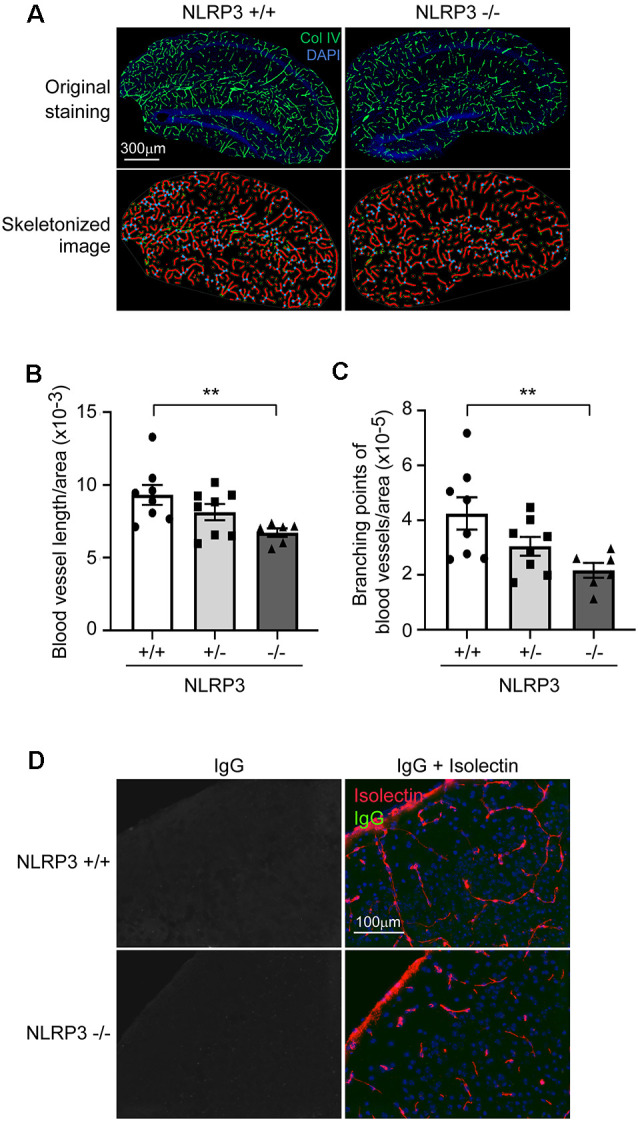
NLRP3 deficiency reduces vasculature in the brain. **(A)** The brains of 9-month-old littermate mice with homozygous (−/−), heterozygous (+/−), and wild-type (+/+) of *nlrp3* gene were stained for collagen type IV (Col IV). The blood vessels in the hippocampus were thresholded and skeletonized. The skeleton representation of vasculature is shown in red and branching points of blood vessels are in blue. **(B,C)** The total length and branching points of blood vessels were calculated and adjusted by area of analysis. One-way ANOVA followed by Bonferroni *post hoc* test, *n* = 8, 8, and 6 for NLRP3 (+/+, +/− and −/−) mice, respectively. **p < 0.01. **(D)** Nine-month-old NLRP3-deficient and wild-type mouse brains were further stained for mouse IgG and isolectin B4. We could not detect mouse IgG in the brain parenchyma.

Pericytes are essential for the development of cerebral circulation. Dysfunction of pericytes reduces vasculature and increases the permeability of BBB (Sweeney et al., 2016; Montagne et al., 2018). We asked whether NLRP3 deficiency affects the structure of cerebral blood vessels. We observed that, in 9-month-old mouse brains, deficiency of NLRP3 significantly reduced the total length and branching points of collagen type IV-positive blood vessels (Figures 2A–C; one-way ANOVA, *p* < 0.05; *n* ≥ 6 per group). The reduction of brain vasculature was dependent on the copies of the NLRP3-encoding gene. However, we did not detect IgG leakage into the brain parenchyma, which suggested that the intactness of BBB in NLRP3-deficient mice was not severely damaged (see Figure 2D).

### NLRP3 Inhibition Attenuates Cell Proliferation in Cultured Pericytes

**Figure 3:**
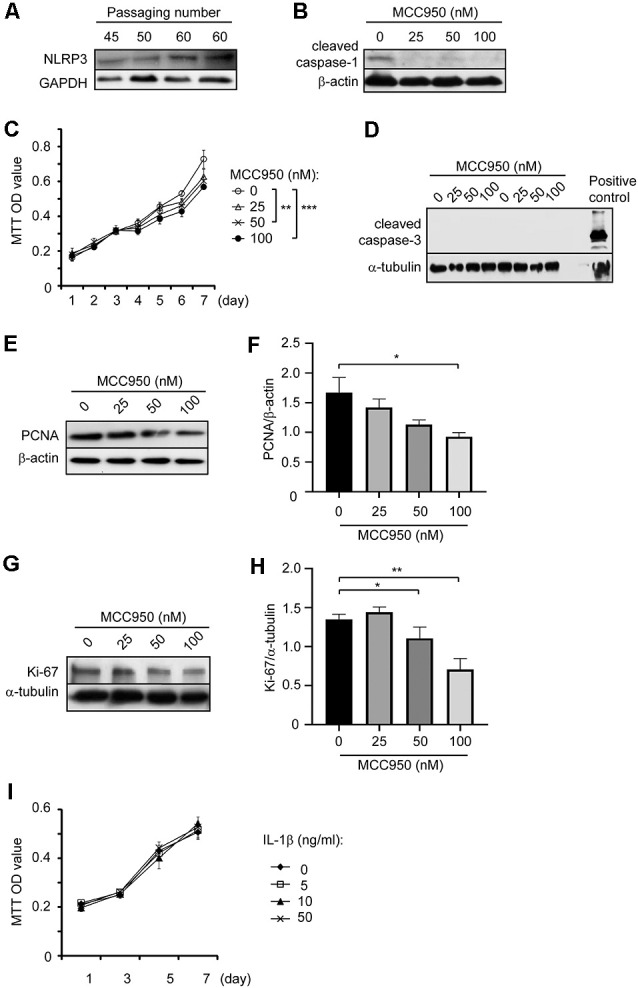
NLRP3 inhibition attenuates cell proliferation in cultured pericytes. **(A)** The cell lysates collected from cultured pericytes with different passaging numbers were detected for NLRP3 with Western blot. **(B)** Pericytes were treated with NLRP3 inhibitor, MCC950, at 0, 25, 50, and 100 nM for 24 h and then detected for cleaved caspase-1. After inhibition of NLRP3, cleaved caspase-1 was nearly undetectable. **(C)** Cultured pericytes were treated with MCC950 with various doses and analyzed for proliferation with MTT assay every day for 7 days. Two-way ANOVA followed by Tukey *post hoc* test, *n* = 4 per group. **p < 0.01 and ***p < 0.001. **(D–H)** Pericytes were cultured and treated with MCC950 at indicated concentrations for 24 h. Cell lysates were detected for cleaved caspase-3, PCNA, and Ki-67 with a quantitative Western blot. As a positive control for cleavage of caspase-3, the brain lysate from neuronal ATG5-deficient mice was used. The result in **(D)** shows one experiment representative of four independent experiments. The inhibition of NLRP3 reduces protein levels of PCNA and Ki-67 in a dose-dependent manner. One-way ANOVA followed by Tukey *post hoc* test, *n* = 3 per group for PCNA, and *n* = 4 per group for Ki-67. *p < 0.05 and **p < 0.01. **(I)** cultured pericytes were further treated with recombinant IL-1β at 0, 5, 10, and 50 ng/ml and analyzed with MTT assay every 2 days for 7 days. Two-way ANOVA analysis did not show the effects of IL-1β on cell proliferation, *n* = 3 per group.

After we observed that NLRP3 deficiency decreased the number of pericytes in the brain, we continued to investigate whether NLRP3 directly regulates the proliferation and death of pericytes. We detected NLRP3 proteins in our cultured pericytes with different passaging numbers (Figure 3A). We also detected cleaved caspase-1 in pericytes, with the remarked reduction of proteins after cells were treated with NLRP3 inhibitor, MCC950 (Figure 3B). Thus, the NLRP3-caspase-1 signaling pathway is active in pericytes and potentially regulates the cellular function. Activated caspase-1 cleaves pro-IL-1β into active IL-1β (Gross et al., 2011). However, we were not able to show that NLRP3 inhibition reduced IL-1β secretion as the IL-1β release from non-activated pericytes was undetectable (data are not shown).

Then, we investigated whether NLRP3 regulates pericyte proliferation. After treating cultured pericytes with MCC950 at different concentrations, we observed that NLRP3 inhibition significantly reduced the conversion of MTT into its colorful product, formazan, in a dose-dependent manner (Figure 3C; two-way ANOVA, *p* < 0.05; *n* = 4 per group). The amount of formazan as shown with OD values is proportional to the number of viable cells. In further experiments, we detected no cleavage of caspase-3 in MCC950-treated cells (Figure 3D), while MCC950 treatments significantly decreased protein levels of both PCNA (Figures 3E,F; one-way ANOVA, *p* < 0.05; *n* = 3 per group) and Ki-67 (Figures 3G,H; one-way ANOVA, *p* < 0.05; *n* = 4 per group), which are two typical protein markers for cell proliferation. Thus, inhibition of NLRP3 suppresses the proliferation of pericytes.

In additional experiments, we treated cultured pericytes with recombinant IL-1β cytokine. IL-1β at 5, 10 and 50 ng/ml did not alter the cell proliferation as measured with MTT assay (Figure 3I; *n* = 3 per group).

### NLRP3 Inhibition Attenuates the Expression of PDGFRβ and CD13 in Cultured Pericytes

**Figure 4:**
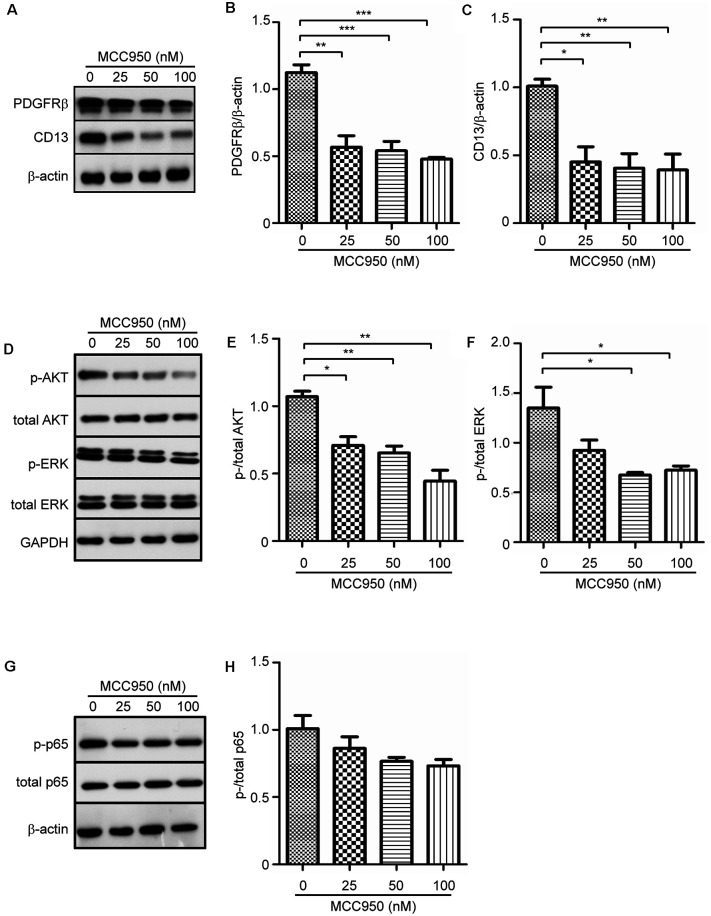
NLRP3 inhibition attenuates protein expression of PDGFRβ and CD13 and inhibits phosphorylation of AKT and ERK in cultured pericytes. Pericytes were cultured and treated with MCC950 at 0, 25, 50, and 100 nM for 24 h. **(A,D,G)** Western blot was used to detect PDGFRβ and CD13, as well as phosphorylated and total protein levels of AKT, ERK, and NFκB p65. **(B,C,E,F)** Inhibition of NLRP3 reduces protein levels of PDGFRβ and CD13 and inhibits phosphorylation of AKT and ERK with a dose-dependent pattern. one-way ANOVA followed by Tukey *post hoc* test, *n* = 4 per group. *p < 0.05, **p < 0.01 and ***p < 0.001. **(H)** Phosphorylation of NFκB p65 is not significantly changed by inhibition of NLRP3. One-way ANOVA, *n* = 3 per group.

PDGFRβ and CD13 are two protein markers of pericytes in the brain, which mediate the physiological and pathophysiological functions of pericytes (Lindahl et al., 1997; Rangel et al., 2007). We observed that treatments with MCC950 inhibited expression of PDGFRβ and CD13 in pericytes in a dose-dependent manner (Figures 4A–C; one-way ANOVA, *p* < 0.05; *n* = 4 per group). To analyze underlying mechanisms, through which NLRP3 drives pericyte differentiation, we detected phosphorylation of AKT, ERK, and NF-κB in MCC950-treated cells. Activation of AKT and ERK is involved in pericyte proliferation and migration (Bonacchi et al., 2001; Yao et al., 2014). We observed that inhibition of NLRP3 reduced the protein levels of both phosphorylated AKT and ERK in a dose-dependent manner (Figures 4D–F; one-way ANOVA, *p* < 0.05; *n* = 4 per group). However, phosphorylation of NF-κB in pericytes was not significantly altered by treatments with MCC950 (Figures 4G,H; one-way ANOVA, *p* = 0.094; *n* = 3 per group).

### IL-1β Increases the Expression of PDGFRβ and CD13 in Cultured Pericytes

**Figure 5:**
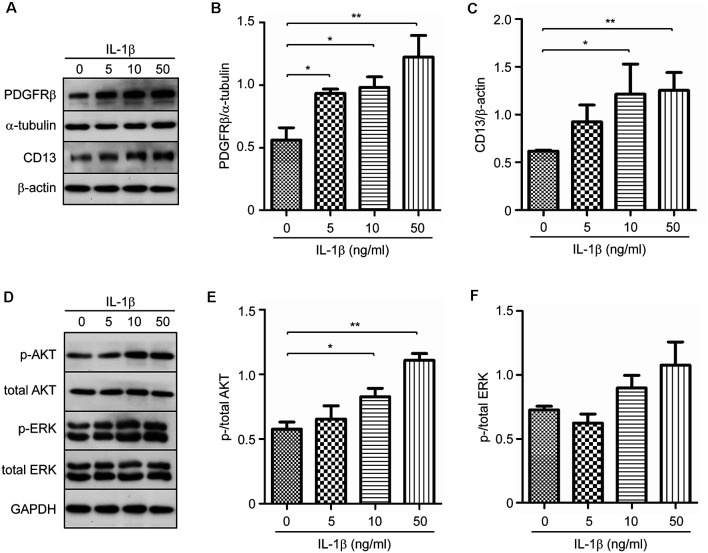
IL-1β increases protein expression of PDGFRβ and CD13 in cultured pericytes. Pericytes were cultured and treated with recombinant human IL-1β at 0, 5, 10, and 50 ng/ml for 24 h. **(A,D)** Western blot was used to detect PDGFRβ and CD13, as well as phosphorylated and total protein levels of AKT and ERK. **(B,C,E,F)** Stimulation of IL-1β increases protein levels of PDGFRβ and CD13, and activates phosphorylation of AKT, but not ERK, in a dose-dependent manner. One-way ANOVA followed by Tukey *post hoc* test, *n* = 3 per group. *p < 0.05 and **p < 0.01.

Although IL-1β did not increase pericyte proliferation (Figure 3I), we hypothesized that IL-1β might affect the differentiation of pericytes. We treated cultured pericytes with IL-1β at different concentrations. IL-1β did increase the protein expression of PDGFRβ and CD13 also with a concentration-dependent pattern (Figures 5A–C; one-way ANOVA, *p* < 0.05; *n* = 3 per group). As potential mechanisms mediating effects of IL-1β activation, we observed that IL-1β treatments significantly increased phosphorylation of AKT but not of ERK (Figures 5D–F; one-way ANOVA, *p* < 0.05; *n* = 3 per group).

### Inhibition of AKT Suppresses Expression of PDGFRβ and CD13 in Cultured Pericytes

**Figure 6:**
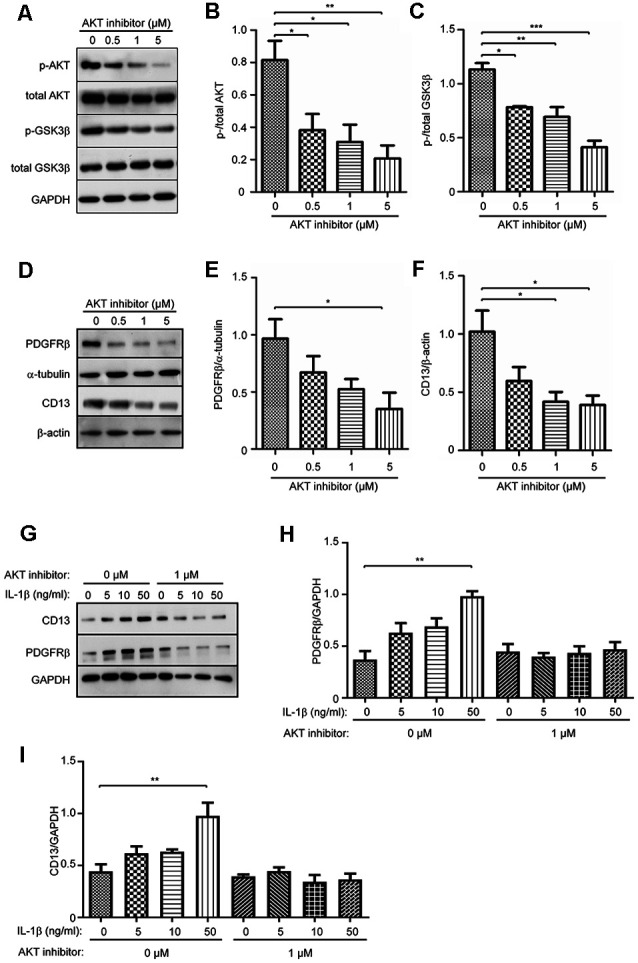
Inhibition of AKT signaling pathway suppresses protein expression of PDGFRβ and CD13 and abolishes the effect of IL-1β in cultured pericytes. Pericytes were cultured and treated with AKT inhibitor at 0, 0.5, 1, and 5 μM for 24 h. **(A–C)** Western blot was used to detect phosphorylated and total AKT, and GSK3β, which shows that phosphorylation of both AKT and GSK3β was significantly inhibited by treatments with AKT inhibitors. One-way ANOVA followed by Tukey *post hoc* test, *n* = 4 per group. **(D–F)** The cell lysate from AKT inhibitor-treated pericytes were detected for protein levels of PDGFRβ and CD13 with a quantitative Western blot. The inhibition of AKT decreases protein levels of PDGFRβ and CD13 in a dose-dependent manner. One-way ANOVA followed by Tukey *post hoc* test, *n* = 3 per group. *p < 0.05, **p < 0.01 and ***p < 0.001. **(G–I)** Cultured pericytes were pre-treated with 1 μM AKT inhibitor and then incubated with IL-1β at 0, 5, 10, and 50 ng/ml, in the presence of AKT inhibitor for 24 h. AKT inhibition abolished the effect of IL-1β on the up-regulation of PDGFRβ and CD13 expression. one-way ANOVA followed by Tukey *post hoc* test, *n* = 3 per group. **p < 0.01.

As activation of AKT in pericytes was suppressed by NLRP3 inhibition but enhanced upon IL-1β activation, we supposed that AKT signaling plays a key role in the differentiation of pericytes. We treated pericytes with AKT inhibitors at 0, 0.5, 1, and 5 μM. Phosphorylation of AKT and phosphorylation of GSK3β, a kinase down-stream to AKT, were both reduced (Figures 6A–C; one-way ANOVA, *p* < 0.05; *n* = 4 per group), which verified the successful inhibition of AKT signaling. With such an inhibition, expression of both PDGFRβ and CD13 was significantly down-regulated in a dose-dependent manner (Figures 6D–F; one-way ANOVA, *p* < 0.05; *n* = 3 per group).

In the following experiments, we treated cultured pericytes with IL-1β at different concentrations in the presence and absence of 1 μM AKT inhibitor. Without co-treatment of AKT inhibitor, IL-1β activation did increase the expression of PDGFRβ and CD13 in pericytes (Figures 6G–I; one-way ANOVA, *p* < 0.05; *n* = 3 per group), which corroborated our previous experiments (see Figure 5). Interestingly, inhibition of AKT abolished the effect of IL-1β on the up-regulation of PDGFRβ and CD13 proteins in pericytes (Figures 6G–I; one-way ANOVA, *p* > 0.05; *n* = 3 per group).

## Discussion

Pericytes play a central role in regulating microvascular circulation and BBB function in the brain (Sweeney et al., 2016). Our study demonstrated that the deletion of NLRP3 under physiological conditions decreases the coverage of pericytes and protein levels of PDGFRβ and CD13 in cerebral blood vessels. PDGFRβ and CD13, together with neural/glial antigen-2 and CD146 are expressed in capillary-associated pericytes, and often used as protein markers of brain pericytes (Smyth et al., 2018). PDGFRβ and CD13 also trigger proliferation and migration of pericytes after stimulation with angiogenesis-associated growth factors (Lindahl et al., 1997; Rangel et al., 2007). Thus, the reduction of CD13 and PDGFRβ represents not only the loss of pericytes but also the dysfunction of pericytes in the brain. Indeed, we observed that the deletion of NLRP3 reduces the vasculature in the brain, which corroborates a recent observation that the dysfunction of pericytes decreases the length of cerebral blood vessels in PDGFRβ-mutated mouse brain (Montagne et al., 2018). Our study suggests that NLRP3 is essential in the maintenance of functional pericytes in healthy brains. We did not observe the leakage of IgG from serum into brain parenchyma in NLRP3-deficient mice. However, we could not exclude the impairment in the ultrastructure of BBB, which might be caused by the lack of NLRP3.

NLRP3-contained inflammasome activates caspase-1 and produces active IL-1β (Gross et al., 2011). MyD88 mediates inflammatory activation after the challenges of TLRs ligands and IL-1β (O’Neill and Bowie, 2007). We observed that deficiency of either NLRP3 or MyD88 decreases protein levels of PDGFRβ and CD13 in the mouse brain. We supposed that NLRP3 drives a basal inflammatory activation in pericytes and promotes pericyte survival, although it was difficult to detect the secretion of IL-1β from pericytes in the brain. In cultured pericytes, we did observe that caspase-1 is activated and inhibition of NLRP3 attenuates phosphorylation of multiple inflammation-related kinases, such as AKT and ERK, and perhaps also NFκB (*p* = 0.094), which is correlated with decreased cell proliferation and PDGFRβ and CD13 expression. Moreover, treatments with IL-1β increase PDGFRβ and CD13 expression in our cultured pericytes. It is consistent with a report that TNF-α at 10 ng/ml promotes cultured pericytes to proliferate and migrate (Tigges et al., 2013). Thus, it is not surprising that angiogenesis is activated with pericyte proliferation in inflammatory lesion sites of multiple sclerosis (Girolamo et al., 2014). However, we observed that IL-1β treatments did not increase pericyte proliferation, which suggests that the regulation of pericyte proliferation by NLRP3 might not depend on IL-1β production. During early wound healing, NLRP3 facilitates angiogenesis; and the production of IL-1β was not always necessary for this repair process, either (Weinheimer-Haus et al., 2015).

As there are no pericyte-specific NLRP3-knockout mice available, we had to work on animals with an overall deficiency of NLRP3. We know that NLRP3 is expressed also in non-pericyte brain cells, i.e., microglia (Heneka et al., 2013). We cannot exclude the possibility that NLRP3 deficiency in microglia decreases the coverage of pericytes in brain vessels. Microglia, located at the perivascular space, is the major component of the neurovascular unit. In response to systemic inflammation, microglia protect BBB integrity in an initial phase by expressing tight-junction protein Claudin-5 and turn to damage BBB through phagocytizing astrocytic end-feet after the inflammation is sustained (Haruwaka et al., 2019). However, the regulation of BBB function and especially pericyte activation by blood vessel-associated microglia under physiological conditions has not been fully explored.

It should be noted that NLRP3 might not fully offer protective effects on pericytes when the inflammatory activation surrounding pericytes is uncontrolled. In the AD brain, NLRP3-contained inflammasome is activated (Heneka et al., 2013), whereas, pericytes are impaired and lost (Sengillo et al., 2013; Nation et al., 2019). There must be other mechanisms, which damage pericytes, compensating for the protective effects of NLRP3 activation. For example, brain-delivered neurotrophic factor (BDNF) drops down and the activation of the BDNF receptor, TrkB, is impaired in the AD brain (Tanila, 2017). TrkB signaling regulates pericyte migration. The deletion of TrkB in pericytes reduces pericyte density and causes abnormal vasculogenesis in the heart (Anastasia et al., 2014). The deletion of NLRP3 in APP or Tau-transgenic mice was reported to rescue neuronal functions and shift microglial activation from pro-inflammatory to anti-inflammatory profile (Heneka et al., 2013; Ising et al., 2019). Unfortunately, the microvascular circulation in NLRP3-deficient AD mice has not been investigated.

AKT is a known kinase to regulate cell survival, proliferation, and angiogenesis in response to extracellular signals (Manning and Cantley, 2007). AKT activation prevents pericyte loss in diabetic retinopathy (Yun et al., 2018). In our experiments, AKT phosphorylation is reduced by NLRP3 inhibition but enhanced by IL-1β activation. Inhibition of AKT directly down-regulates the expression of PDGFRβ and CD13 and also abolishes the effect of IL-1β to elevate the protein levels of PDGFRβ and CD13 in pericytes. Thus, AKT activation might mediate the protective effects of NLRP3 in pericytes. As PDGFRβ activation induces phosphorylation of AKT (Lehti et al., 2005), PDGFRβ and AKT even activate each other and form positive feedback to maintain the healthy pericytes in the brain.

In summary, our study suggested that NLRP3 activation maintains healthy pericytes in the brain through activating AKT signaling pathway. In the following studies, we are evaluating potential cerebral vascular impairment in AD mice with both genetic and pharmacological inhibitions of NLRP3. The potential adverse effects of NLRP3 inhibition on pericyte function and microcirculation should be considered when NLRP3 inhibitors are administered to treat AD or other inflammatory disorders.

## Data Availability Statement

The original contributions presented in the study are included in the article, further inquiries can be directed to the corresponding author.

## Ethics Statement

The animal study was reviewed and approved by Landesamt für Verbraucherschutz, Saarland.

## Author Contributions

YL designed the study and wrote the manuscript. WQ, QL and QT conducted experiments, acquired data and analyzed data. TF provided pericyte cell line. DL and KF supervised the study. All authors contributed to the article and approved the submitted version.

## Conflict of Interest

The authors declare that the research was conducted in the absence of any commercial or financial relationships that could be construed as a potential conflict of interest.

## Footnotes

^1^https://imagej.nih.gov/ij/

^2^https://ccrod.cancer.gov/confluence/display/ROB2/

## Abbreviations

Aβ: amyloid β peptide
AD: Alzheimer’s disease
APP: Alzheimer’s precursor protein
BBB: blood-brain barrier
BDNF: brain-delivered neurotrophic factor
HBPC: human primary brain vascular pericytes
HBSS: Hanks’ balanced salt solution
IL-1β: interleukin-1β
LPS: lipopolysaccharide
MyD88: myeloid differentiation primary response 88
NLRP: NACHT, LRR and PYD domains-containing protein
PCNA: proliferating cell nuclear antigen
PDGFRβ: platelet-derived growth factor receptor β
TLR: Toll-like receptor
TNF-α: tumor necrosis factor α.
